# Cobalt chloride inhibits tumor formation in osteosarcoma cells through upregulation of HIF-1α

**DOI:** 10.3892/ol.2013.1127

**Published:** 2013-01-10

**Authors:** BO ZHANG, WEICHUN GUO, LING YU, FU’AN WANG, YONG XU, YANG LIU, CHENGXIAO HUANG

**Affiliations:** Department of Orthopedics, Renmin Hospital, Wuhan University, Wuhan 430060, P.R. China

**Keywords:** hypoxia, CoCl_2_, osteosarcoma, HIF-1α

## Abstract

The exact effect of hypoxia on cancer development is controversial. The present study investigates the ability of osteosarcoma to form tumors in the hypoxic microenvironment induced by CoCl_2_. MG63 human osteosarcoma cells were cultured with different concentrations (0, 150 and 300 *μ*M) of CoCl_2_ for 24 h to simulate hypoxia *in vitro*. The expression of hypoxia-inducible factor (HIF)-1α was analyzed by western blotting. The proliferation and drug resistance of MG63 cells were examined using the CCK-8 assay, the apoptosis rate was detected by flow cytometry, the ability to form spheroids was assessed by a sarcosphere culture system and invasiveness was determined by a vertical invasion assay. A transplantation assay was used to evaluate the ability to form tumors *in vivo*. Our results showed that the proliferation of MG63 cells was inhibited by treatment with CoCl_2_, while no effect on drug toxicity was observed. The apoptotic rate was increased in a dose-dependent manner, the ability to form sarcospheroids was suppressed, the invasiveness was inhibited and the expression of HIF-1α was upregulated following CoCl_2_ treatment. We also found that the ability to form tumors *in vivo* was inhibited. In conclusion, we provide strong evidence that CoCl_2_ has the ability to inhibit osteosarcoma development; the mechanism may be related to the hypoxic microenvironment and HIF-1α may be a critical regulatory factor.

## Introduction

Osteosarcomas are primary malignant tumors of the bone which are now believed to be derived from malignant mesenchymal stem cells (MSCs) (1). The tumors mostly occur in the metaphyses of long bones, especially the distal femur, the proximal tibia and the proximal humerus (2). The World Health Organization (WHO) classifies conventional osteosarcoma into three main subtypes: osteoblastic, chondroblastic and fibroblastic (3). In the last 40 years, the application of adjuvant chemotherapy has improved the survival of osteosarcoma patients. However, the 5-year survival rate is only ∼65% and the rates after recurrence or metastasis are worse, only ∼30% (4). Without formal treatment, osteosarcoma migrates to other tissues, most commonly to the lung, in 6 months to one year and leads to mortality (5). Therefore, the effective treatment of osteosarcomas is worthy of study.

The tumor microenvironment is different from the normal environment of the body in physical and chemical properties, including hypoxia and low pH (6). In 1955, Thomlinson first noted that a number of malignant tumor tissues have hypoxic areas (7). Hypoxia-inducible factor-1 (HIF-1) was then identified by Semenza when he studied the expression of the erythropoietin gene induced by hypoxia (8). HIF-1 is a heterodimeric transcription factor composed of two subunits, oxygen-dependent HIF-1α and constitutively expressed HIF-1β (9). HIF-1α has been reported to activate the transcription of a set of genes which contribute to tumor aggressiveness, including VEGF, ENOI, TGF-α and CXCR4. By contrast, HIF-1α is also thought to inhibit tumor growth; for example, Carmeliet *et al* observed that tumors derived from HIF-1-deficient embryonic stem (ES) cells formed larger tumors compared with wild-type (HIF-1α+/+) (10).

To investigate the effect of the hypoxic microenvironment on osteosarcoma, we used CoCl_2_ to simulate a hypoxic micro-environment (11). There were two reasons why we selected CoCl_2_ as the hypoxia-inducing agent. Firstly, Co^2+^ replaces Fe^2+^ in hemoglobin, forming deoxygenated hemoglobin. Secondly, Co^2+^ inhibits HIF-lα aryl hydrocarbon-hydroxylase activity to reduce HIF-lα degradation (12). Therefore, the features of CoCl_2_-simulated hypoxia are similar to those of the *in vivo* hypoxic microenvironment. We treated a human osteosarcoma cancer cell line (MG63) with CoCl_2_ to stimulate hypoxia *in vitro*. Under the hypoxic conditions, we observed the characteristics of the cells, including proliferation, drug resistance, apoptosis and tumor formation, by CCK-8, flow cytometry (FCM) and sarcosphere system assays, respectively. Our results revealed that CoCl_2_ stimulated a hypoxic microenvironment *in vitro* and inhibited tumor development.

## Materials and methods

### Reagents

The chemicals used were as follows: Fetal bovine serum (Gibco, USA); RPMI-1640 medium (Gibco); 2-(4-indophenyl)-3-(4-nitrophenyl)-5-(2,4-disulphophenyl)-2 H-tetrazolium monosodium salt (CCK-8; Santa Cruz Biotechnology, Santa Cruz, CA, USA); Annexin V-FITC/PI apoptosis detection kit (Santa Cruz Biotechnology); transwell chamber (Corning, USA); ultralow attachment plates (Corning); HIF-1α monoclonal antibody (Santa Cruz Biotechnology); CoCl_2_ (Sigma, St. Louis, MO, USA); FGF (Sigma) and EGF (Sigma).

### Cell line and cell culture

The human osteosarcoma cancer cell line MG63 was purchased from the Shanghai Institute for Biological Sciences of Chinese Academy of Sciences (Shanghai, China). Cells were cultured in DMEM/F12 medium containing 10% fetal bovine serum (FBS), with 1×10^5^ U/l penicillin and 100 mg/l streptomycin, in a humidified atmosphere in a 5% CO_2_ incubator at 37°C.

### CCK-8 assay for the proliferation and drug resistance of MG63 cells

To determine the effect of CoCl_2_ on MG63 cell proliferation and drug resistance, MG63 cells were treated with different concentrations of CoCl_2_ (0, 150 and 300 *μ*M) for 24 h. For the proliferation assay, 1×10^5^ cells were seeded in each well of 96-well culture plates and cultured for 1 to 5 days for CCK-8 incubation. For the drug resistance assay, cells were cultured (5×10^4^ per well) in 96-well plates for 1 day and then treated with increasing concentrations of doxorubicin and methotrexate for 24 h and then underwent CCK-8 incubation. All the cells were incubated with CCK-8 reagent for 1 h at 37°C. The staining intensity in the medium was measured by determining the absorbance at 450 nm.

### FCM analysis for Annexin V and propidium iodide (PI)

MG63 cells were cultured in 6-well plates and treated with different concentrations of CoCl_2_ (0, 150 and 300 *μ*M) for 24 h. After treatment, cells were harvested with 0.25% trypsin and collected by centrifugation at 900 × g for 5 min at room temperature. Cells were washed and re-suspended in PBS and labeled with Annexin V and PI for 20 min. Fluorescence (DNA content) was measured by FCM using standard software.

### Neurosphere/sarcosphere system assays

MG63 cells were cultured in 6-well plates and pretreated with three concentrations of CoCl_2_ (0, 150 and 300 *μ*M) for 24 h. The cells were then plated at a density of 60,000 cells/well in 6-well ultra low attachment plates in B27 medium with the growth factors human EGF (10 ng/ml) and human FGF (10 ng/ml). Fresh aliquots of EGF and FGF were added every other day. After being cultured for 14 days, colonies containing >50 cells were quantitated by inverted phase contrast microscopy.

### Vertical invasion of cells

MG63 cells were cultured in 6-well plates and pretreated with three concentrations of CoCl_2_ (0, 150 and 300 *μ*M) for 24 h. A transwell assay was used to evaluate the vertical invasion of cells. After treatment, the 6-well plates were washed twice with PBS to remove floating cells. The cells were then re-suspended in culture medium without FBS after conventional digestion. Cell suspensions (100 *μ*l; 2.0×10^5^/ml) were added to the upper chamber and complete culture medium was added to the lower chamber. After 24 h, the upper chamber was removed, fixed with 4% paraformaldehyde for 30 min and stained for 15 min with crystal violet. We randomly selected four fields of vision to count the number of cells which had moved to the lower membrane under a microscope, taking the average of the number of vertically migrated cells.

### Western blot analysis

Cells were treated as described above. Protein was extracted from subconfluent cultures using lysis buffer containing 1 mM PMSF and quantified using the BCA method. Aliquots of 40 *μ*g protein from each sample were then resolved using SDS-PAGE and subsequently transferred to PVDF membranes. Membranes were blocked in 5% milk solution and incubated with primary antibody at 4°C overnight. The membranes were then washed and incubated with horseradish peroxidase-conjugated secondary antibody (13). The immunoreactivity was detected by chemiluminescence. Statistical analyses of the western blotting data were performed on the densitometric values obtained with NIH IMAGE 1.61 software.

### Animals and transplantation assay

To determine the *in vivo* tumorigenicity, we established subcutaneous and orthotopic osteosarcoma animal models. A total of 24 male BALB/C nude mice ∼4–6 weeks old were purchased from and maintained at the Wuhan University Center for Animal Experiment (China). The care and use of animals followed the recommendations and guidelines of the National Institutes of Health and was reviewed and approved by the Institutional Animal Care and Use Committee (IACUC; approval number, 2011006). The mice were randomly divided into 0 and 150 *μ*M groups (6 per group) according to the injected cells. The experiments consisted of two parts: orthotopic and subcutaneous injections. Cells in log-phase growth were harvested, washed and re-suspended with PBS, and the BALB/C nude mice were anesthetized. For orthotopic transplantation, 5×10^6^ cells in 0.1 ml PBS were injected into the left distal femoral bone marrow cavities of each mouse. For the subcutaneous transplantation, we injected 0.1 ml PBS with 2×10^5^ cells into the back of the mice. The mice were monitored daily until one month after injection. We compared the size of the xenografted osteosarcoma tissues and the tumor formation rate of the two groups.

### Statistical analysis

Numerical data are expressed as mean ± SD. Statistical analysis was performed by analysis of variance or Student’s t-test using the SPSS 13.0 statistical program (SPSS, Inc., Chicago, IL, USA). P<0.05 was considered to indicate a statistically significant result.

## Results

### Expression of HIF-1α increased following CoCl_2_ treatment

**Figure 1:**
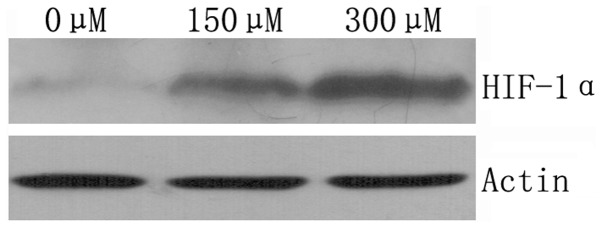
HIF-1α protein expression in MG63 cells following exposure to different concentrations of CoCl_2_. The 120-kDa band corresponds to HIF-1α protein. The expression level of HIF-1α protein after CoCl_2_ exposure was significantly increased in a dose-dependent manner. HIF, hypoxia-inducible factor.

Western blot analysis was performed to verify if exposure of MG63 cells to CoCl_2_ induced HIF-1α expression. As shown in Fig. 1, HIF-1α was undetectable in untreated control cells, while it became detectable in the two other groups.

### Hypoxic microenvironment simulated by CoCl_2_ inhibits MG63 cell proliferation but has no effect on drug resistance

**Figure 2:**
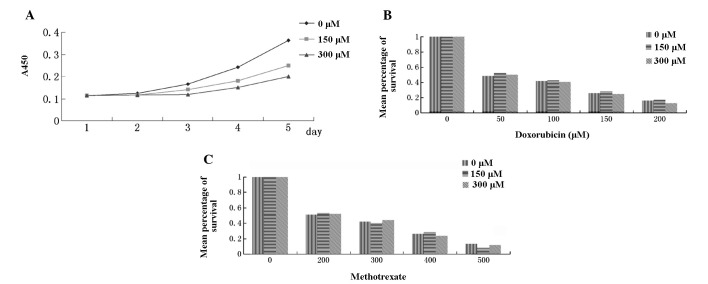
CoCl_2_ inhibits MG63 proliferation and has no effect on drug resistance. (A) Curve for cell proliferation of the three groups at different times. Cells cultured under normoxic conditions proliferated more rapidly than the other two groups. (B and C) There was no significant difference in IC50 following CoCl_2_ exposure.

As shown in Fig. 2A, the growth curve of cells under normoxic conditions showed an ‘S’ shape: the lag phase was 1–2 days (cells grow slowly); the exponential phase of growth was 3–5 days (cells rapidly proliferated). Compared with the normoxic group, the cells of the experimental groups proliferated markedly more slowly. We further investigated the drug resistance properties, but did not find any significant differences following CoCl_2_ treatment (Fig. 2B and C).

### FCM analysis of cell apoptosis induced by CoCl_2_

**Figure 3:**
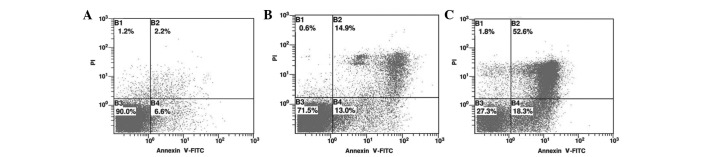
Apoptosis induced by CoCl_2_. Flow cytometry revealed that when the concentration of CoCl_2_ increased, the proportion of apoptotic cells gradually increased. (A) 0 *μ*M; (B) 150 *μ*M; (C) 300 *μ*M CoCl_2_.

Following treatment with different concentrations of CoCl_2_ for 24 h, apoptosis induction was demonstrated using FCM analysis. As shown in Fig. 3, in the normoxic group, cells were almost normal in appearance with rare viable apoptotic cells; while in the experimental group, the rate of apoptotic cells increased with increasing concentrations of CoCl_2_. The rate of apoptosis in the normoxic, 150 and 300 *μ*M CoCl_2_ groups was 6.6, 13.0 and 18.3%, respectively. Furthermore, the proportion of apoptotic cells gradually increased in a dose-dependent manner.

### MG63 sarcospheroid formation was inhibited by CoCl_2_

**Figure 4:**
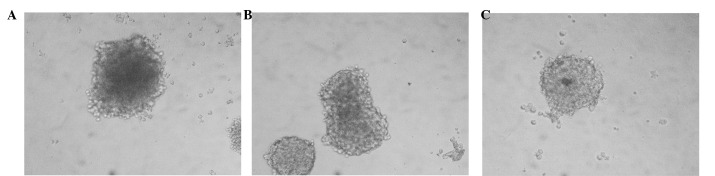
MG63 sarcospheroid formation was inhibited by CoCl_2_. Images of monoclonal sarcospheres formed from self-renewing cells from bone sarcoma. When the concentration of CoCl_2_ increased, the number and size of sarcospheres were gradually reduced. (A) 0 *μ*M; (B) 150 *μ*M; (C) 300 *μ*M CoCl_2_. Magnification, ×400.

All three groups of osteosarcoma cells formed spherical colonies after 10 to 14 days. However, there were marked differences between the groups. In the normoxic group, the mean number of spherical colonies formed was 210±10, whereas that of the 150 *μ*M group was 150±5 and that of the 300 *μ*M group was 70±7 (P<0.05). As shown in Fig. 4, the spherical colonies of the normoxic group were markedly bigger than those of the other two groups. Furthermore, the number and size of the spherical colonies gradually decreased in a dose-dependent manner.

### Inhibition of vertical invasion by CoCl_2_

**Figure 5:**
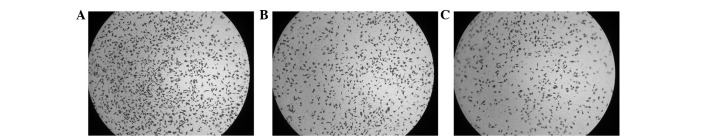
Inhibition of vertical invasion by CoCl_2_. CoCl_2_ markedly inhibits the invasive ability of MG63 cells. (A) 0 *μ*M; (B) 150 *μ*M; (C) 300 *μ*M CoCl_2_. Magnification, ×100/

In the hypoxic group, the number of cells which crossed the extracellular matrix (ECM) gel-coated filter was markedly lower than that in the normoxic group. In addition, we found that at higher concentrations of CoCl_2_, fewer cells crossed the ECM gel-coated filter (Fig. 5).

### Hypoxic microenvironment inhibits tumor formation

**Figure 6:**
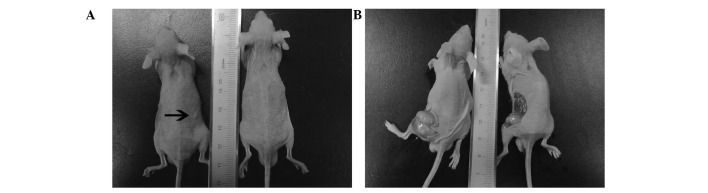
Hypoxic microenvironment inhibits tumor formation. (A) Subcutaneous injection. The incidence of tumor formation was different between the 0 and 150 *μ*M groups. (B) Orthotopic injection. The size of xenografted osteosarcoma tissues was different between the 0 and 150 *μ*M groups. After CoCl_2_ treatment, MG63 sarcospheroid formation was inhibited *in vivo*.

For the subcutaneous transplantation, we found that the 0 *μ*M group formed xenografted osteosarcoma tissues at rate of 100%, however, the 150 *μ*M group rarely formed the tissues. For the orthotopic transplantation, the 0 *μ*M group formed markedly bigger tissues than the 150 *μ*M group. At the end of the assay, the mean volume of the xenografted osteosarcoma tissues in the 0 *μ*M group was 1.24±0.25 cm^3^ and that of the 150 *μ*M group was 0.84±0.2 cm^3^ (P<0.05) (Fig. 6).

## Discussion

Increasing evidence has demonstrated that intratumoral hypoxia may promote invasive growth and metastasis (14). HIF-1α is a key molecule in the hypoxic response (15) and has been found to be overexpressed in ∼70% of tumors (16). However, whether HIF-1α promotes tumor cell apoptosis or has anti-apoptotic affects is controversial. Certain studies have indicated that under hypoxic conditions, the transcriptive activity of HIF-1α was increased, and this in turn enhanced the expression of downstream genes, including VEGF, FGF and TGF-β (17,18). Thus, HIF-1α acts as a positive regulator of tumor development (19). Other studies have reported that HIF-1α upregulates VEGF and GLUT1 to make tumor cells resistant to apoptosis (20). In the present study, we demonstrated that CoCl_2_ simulated a hypoxic microenvironment successfully in MG63 cells. The expression level of HIF-1α was markedly upregulated in the hypoxic microenvironment in a dose-dependent manner. This result is in accordance with those of previous studies using other tumor cell lines (21,22). By contrast, the CCK-8 assay and FCM analysis revealed that CoCl_2_ inhibited the proliferation of MG63 cells and promoted apoptosis, and the effect was enhanced with the increased CoCl_2_ concentration, which shows that CoCl_2_ has the ability to inhibit osteosarcoma growth. Our data are consistent with those reported by Dai *et al*(23). It has also been reported that HIF-1α promotes apoptosis through the PI3K/Akt (24) or ERK 1/2 (25) pathways.

Cell invasive ability is a significant aspect of cancer progression which begins from the migration of tumor cells into contiguous tissues and the dissolution of the ECM. Osteosarcoma has a high tendency to metastasize, especially to the lung. Tumor hypoxia is believed to be correlated with increased metastatic potential, via the regulation of αvβ3 integrin expression and promotion of tumor invasion by the tyrosine kinase receptor MET (26). We thus used a transwell invasion assay to detect whether hypoxia affects the ability of MG63 cells to metastasize. In the process of collecting cells, we removed the floating (dead) cells. We found that CoCl_2_ caused a marked inhibition of invasive ability, which strongly supports the hypothesis that the hypoxic microenvironment is involved in deregulating invasion and metastasis. This was opposite from the findings of previous studies, in which hypoxic conditions elicited tumor cell phenotypes with higher migratory and invasive capacities (27,28).

Previous studies have demonstrated that tumors are composed of heterogeneous populations of cells that differ in their apparent state of self-renewal and differentiation. A subset of the cancer cell population, cancer stem cells, may play important roles in tumorigenesis, metabasis, drug resistance and recurrence (29). The existence of cancer stem cells in tumors is now considered to be the source of tumor initiation and poor prognosis (30). Gibbs *et al* first demonstrated the existence of a small subpopulation of self-renewing bone sarcoma cells that were capable of forming suspended spherical clonal colonies, called ‘sarcospheres’, in anchorage-independent serum-starved conditions (31). Fujii *et al* next demonstrated the existence of these cancer stem cells in MG63 cells. The authors found that certain MG63 cells were also able to form suspended spherical colonies; furthermore, they demonstrated that these MG63 cells showed strong resistance to doxorubicin and cisplatin (32). In the present study, we found that when the concentration of CoCl_2_ increased, the ability of osteosarcoma cells to form sarcospheres was diminished. Therefore, we speculate that CoCl_2_ reduces the ability of the cells to self-renew and promotes the differentiation of cancer stem cells in MG63 cells, inhibiting osteosarcoma carcinogenesis.

Borenstein *et al* used the mammary tumor cell line LMM3 treated with CoCl_2_ for 24 h to detect changes in the *in vivo* growth kinctics. The authors found that the tumors formed by hypoxic cells grew larger than those of controls; moreover, histological examination revealed that control tumors invaded the dermis and epidermis and induced areas of ulceration (33). The results of histological examination were in accordance with those of the present study, but it is unclear what changed the tumorigenic ability in MG63 cells treated with CoCl_2_. Therefore, we further tested the tumorigenic ability of MG63 cells *in vivo*. In the present study, the results showed some differences compared with Borenstein *et al*’s. The orthotopic and subcutaneous transplantations showed that the ability to form tumors was markedly diminished in the CoCl_2_-treated group. This may be due to the different sources of the tumors. However, this is consistent with the result of our neurosphere/ sarcosphere system assays.

In conclusion, the present study provides evidence that the hypoxic microenvironment induced by CoCl_2_ inhibits osteosarcoma development, including inhibiting proliferation, promoting apoptosis, suppressing invasion and eliminating the ability to self-renew. Although there is little information concerning the application of CoCl_2_ in osteosarcoma therapy, we suggest that CoCl_2_ may be used as an antitumor drug, especially in osteosarcoma. However, further investigation into the precise mechanism is required.
